# The Impact of Hyaluronan on Tumor Progression in Cutaneous Melanoma

**DOI:** 10.3389/fonc.2021.811434

**Published:** 2022-01-21

**Authors:** Piia Takabe, Hanna Siiskonen, Aino Rönkä, Kirsi Kainulainen, Sanna Pasonen-Seppänen

**Affiliations:** ^1^ Institute of Biomedicine, University of Eastern Finland, Kuopio, Finland; ^2^ Department of Clinical Pathology, Kuopio University Hospital, Kuopio, Finland; ^3^ Department of Oncology, Kuopio University Hospital, Kuopio, Finland

**Keywords:** melanoma, hyaluronan, skin, UV radiation, tumor microenvironment

## Abstract

The incidence of cutaneous melanoma is rapidly increasing worldwide. Cutaneous melanoma is an aggressive type of skin cancer, which originates from malignant transformation of pigment producing melanocytes. The main risk factor for melanoma is ultraviolet (UV) radiation, and thus it often arises from highly sun-exposed skin areas and is characterized by a high mutational burden. In addition to melanoma-associated mutations such as BRAF, NRAS, PTEN and cell cycle regulators, the expansion of melanoma is affected by the extracellular matrix surrounding the tumor together with immune cells. In the early phases of the disease, hyaluronan is the major matrix component in cutaneous melanoma microenvironment. It is a high-molecular weight polysaccharide involved in several physiological and pathological processes. Hyaluronan is involved in the inflammatory reactions associated with UV radiation but its role in melanomagenesis is still unclear. Although abundant hyaluronan surrounds epidermal and dermal cells in normal skin and benign nevi, its content is further elevated in dysplastic lesions and local tumors. At this stage hyaluronan matrix may act as a protective barrier against melanoma progression, or alternatively against immune cell attack. While in advanced melanoma, the content of hyaluronan decreases due to altered synthesis and degradation, and this correlates with poor prognosis. This review focuses on hyaluronan matrix in cutaneous melanoma and how the changes in hyaluronan metabolism affect the progression of melanoma.

## Introduction

Cutaneous melanoma is a skin cancer that develops from melanocytes. Melanocytes reside in the basal layer of epidermis and synthesize melanin pigment that is transferred in melanosomes to the surrounding keratinocytes to form a protective shield against the harmful effects of UV. Family history, genetic factors, number of nevi and actinic damage have been identified as risk factors for melanoma (1). Solarium is one source for recreational UV exposure, but the impact of moderate solarium use on melanoma risk is still controversial (2). However, The World Health Organization has listed solar radiance as a carcinogen already in 2009 (3).

The American Cancer Society estimates that over 106 000 new melanomas will be diagnosed and over 7000 melanoma-related deaths will occur during this year (2021) (4). In other countries, also in northern countries such as Finland, almost 2000 new melanomas and over 200 melanoma-related deaths occur annually, causing the incidence of 32.74/100 000 persons (5). Melanoma affects mostly people with a light skin type (Caucasians) and the incidence is 27.5/100,000 people compared to the incidence of 1.1/100,000 among people with a dark skin type (6). UV radiation is the most critical risk factor for melanoma development due to its high mutagenic properties. The rate of basal mutations in melanoma (100 mutations/Mb of entire exome) is higher in comparison to other cancers (7).

Hyaluronan is a glycosaminoglycan found in the extracellular space of most tissues, but it also forms a pericellular coat surrounding individual cells. Hyaluronan is involved in many biological and pathophysiological processes such as tissue homeostasis and integrity, fertilization, wound healing, inflammation, angiogenesis, and cancer. Hyaluronan is mainly produced as a high molecular weight (HMW) chain by hyaluronan synthases (HAS) and degraded to lower molecular weight (LMW) fragments or oligosaccharides by hyaluronidases (HYAL) or environmental factors such as reactive oxygen species (ROS) or UV radiation. The effects of hyaluronan on immune, non-malignant and malignant cells are size dependent. HMW hyaluronan has been shown to be relatively inert, and is more physiological, while its degradation products are more abundant during inflammation and angiogenesis, as reviewed in (8–11), and have been speculated to be biologically active and to modulate the tumor microenvironment (TME) (12).

The role of hyaluronan in cancers has been studied broadly. In cancers originating from simple epithelium such as breast and prostate cancer, the increase of hyaluronan in the tumor and the stroma indicates aggressive type of cancer and poor prognosis (13–17). In melanoma and cancers originating from stratified epithelium, however, poor prognosis is associated with the reduced amount of hyaluronan. Benign nevi and melanoma *in situ* contain abundant amounts of hyaluronan whereas in deep and metastatic melanomas the hyaluronan content is scarce. This is due to the reduced expression of HAS1 and 2 and the increased expression of HYAL2 in melanoma cells (18). The loss of HAS2 has shown to be associated with the poor prognosis and recurrence of melanoma (19). The role of hyaluronan in melanoma development and its progression is the focus of this review.

## Cutaneous Melanoma

### The Structure of Skin and Melanocytes

Skin covers the human body and comprises over 20% of the body weight. In addition to providing a physical external barrier and insulation, it is also involved in the process of thermoregulation, pain sensation and chemical messaging (20). Keratinocytes are the main cell type in the avascular epidermis. Epidermis also contains pigment-producing melanocytes, which locate in the basal cell layer, Merkel cells, which are mechanoreceptors and Langerhans cells, which are antigen-presenting dendritic cells (21). The dermal compartment is separated from the epidermis by the basement membrane, and it contains blood and lymphatic vessels, nerve endings for mechanoreception, adnexal structures, and an abundant extracellular matrix (ECM). The ECM is composed of collagen, elastin, glycoproteins like fibronectin, proteoglycans like versican and glycosaminoglycans like hyaluronan, which all are mainly produced by fibroblasts. The subcutis or hypodermis resides deep in the dermis attaching the skin to the underlying skeletal muscles. Due to the structure and organization of the dermis it provides strength and support to the entire skin (20, 22).

In addition to skin, the neural crest-derived melanocytes can be found in hair follicles and in the uvea of the eye (23). Melanocytes produce mainly two types of melanin pigment, eumelanin and pheomelanin. Eumelanin (brown-black pigment) is produced during UV exposure to protect the skin from oxidative (UVA) and direct (UVB) DNA damage. Pheomelanin (yellow-red) is enriched among individuals with red hair and pale skin tone, who lack eumelanin synthesis and are more prone to develop melanoma due to spontaneous reactive oxygen species (ROS) production (24). The dendritic nature of melanocytes with their long axial protrusions and large surface area helps them to transfer melanin granules to adjacent keratinocytes for photoprotection. Melanocytes also have immunomodulatory properties in skin (25).

**Figure 1:**
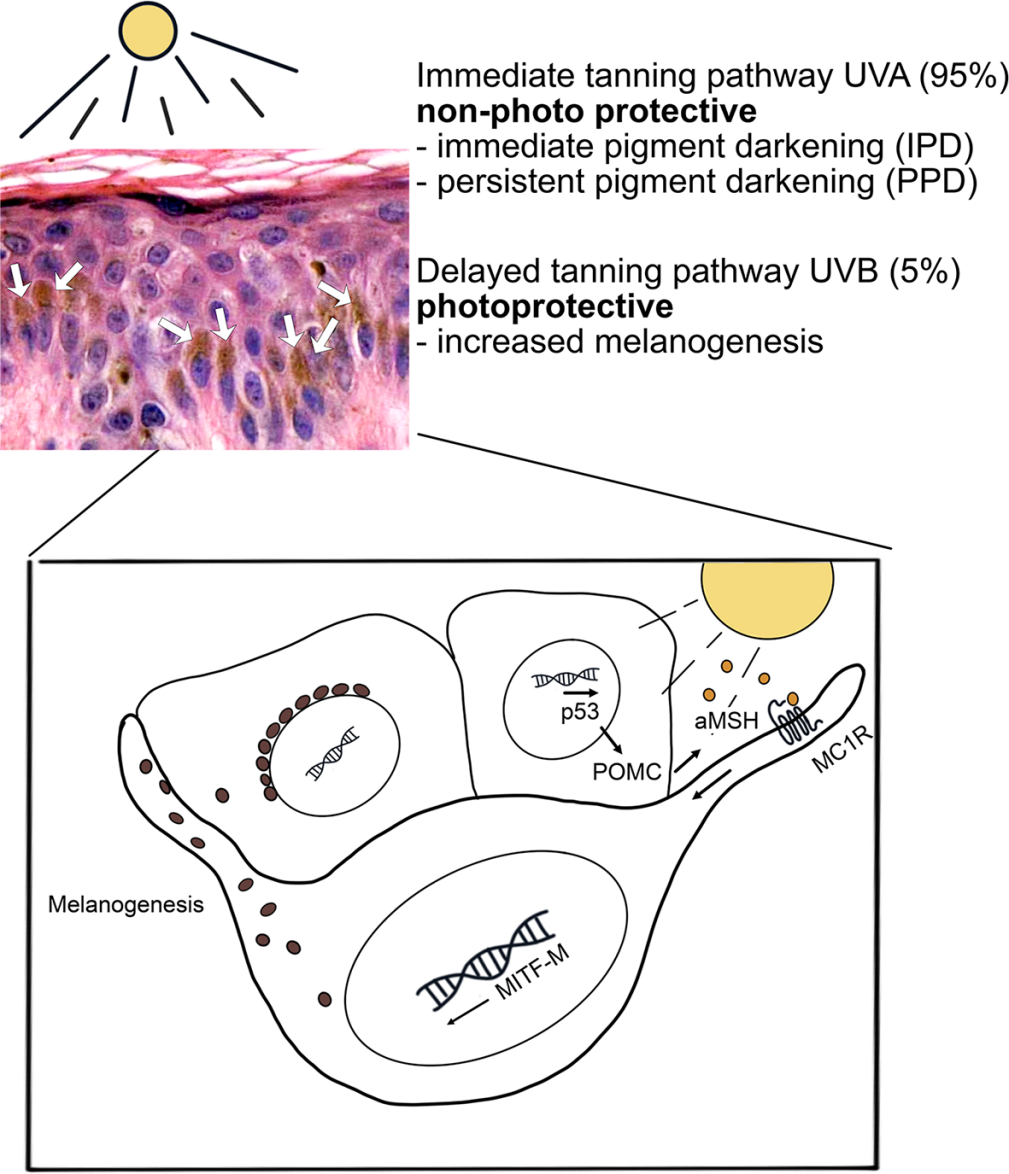
UV-induced tanning pathways in melanocytes. UV radiation induces two tanning pathways in melanocytes. The UVA-induced immediate tanning pathway is non-photoprotective and leads to persistent pigment darkening. The photoprotective tanning pathway is activated firstly in keratinocytes by UVB. In keratinocytes, activated p53 pathway induces α-MSH production and secretion. Secreted α-MSH leads to MC1 receptor-mediated MITF-M activation in melanocytes and increase in melanogenesis. Mature melanosomes are transported into keratinocytes to form a protective perinuclear cap against UVR. Hematoxylin eosin staining of skin epidermis, several basal keratinocytes show accumulated melanin granules (brown color, white arrows) above the cell nuclei.

UV exposure causes the activation of two pathways in melanocytes, namely the immediate tanning pathway by UVA and the delayed tanning pathway by UVB (**Figure 1**). The immediate tanning pathway is non-photoprotective and leads to immediate and persistent pigment darkening whereas the delayed tanning pathway is photoprotective *via* increasing melanogenesis. In keratinocytes, UVB increases p53-mediated transcription of POMC (pro-opiomelanocortin) which is post-translationally processed leading to α-MSH (α-melanocyte stimulating hormone), ACTH (adrenal corticotropin hormone) and opioid peptide β-endorphin production. α-MCH is secreted from keratinocytes to the adjacent melanocytes and, then by activation of MC1R (melanocortin 1 receptor), it induces MITF-M (microphtalmia-associated transcription factor isoform M) transcription and melanosome production. After the maturation of melanosomes, they are transported into basal cell layer keratinocytes to form a protective perinuclear cap against harmful effects of UVR (26). Other mechanisms activated in melanocytes in response to UV-related damage are unfolded protein response (UPR) and integrated stress response (ISR). These mechanisms foster the renewal of homeostasis by activating factors with antioxidant activity, such as NRF2 (nuclear factor erythroid 2-related factor 2) that tackles the damaging effects of ROS. Failure in these repair mechanisms leads to the survival of damaged cells, their adaptation to the environment and promotion of carcinogenesis (27, 28).

### The Classification of Melanoma

Cutaneous melanoma was earlier classified to four major clinical subtypes: superficially spreading melanoma (SSM), nodular melanoma (NM), acral lentiginous melanoma (ALM) and lentigo maligna melanoma (LMM) (29). Interestingly, the World Health Organization recently published a new classification based on the evolutionary pathways of each subtype. The new classification divides melanomas with relation to sun exposure (30). Melanoma subtypes associated with cumulative solar damage (CSD) are superficially spreading melanoma (low-CSD melanoma), lentigo maligna melanoma (high-CSD melanoma) and desmoplastic melanoma. Melanoma subtypes that are not consistently associated with CSD are Spitz melanoma, acral melanoma, mucosal melanoma, melanoma arising in congenital or blue nevi and uveal melanoma. Nodular melanoma constitutes the third group in the new classification (30, 31). Melanomas associated with CSD are more common among Caucasians while non-CSD (without cumulative solar damage) melanomas affect more often the non-Caucasian population (32). High frequency of specific BRAF mutations and patients’ younger age are characteristic for non-CSD. CSD melanomas arise in areas with chronic sun exposure such as face, ears, neck and lower extremities and are associated with high mutational burden. CSD is usually diagnosed in elder people (>60 years) associating with solar elastosis and other non-melanoma skin cancers (31).

SSMs emerge at younger age in comparison to NM or LMM. Typical manifestation of SSM is a flat, slowly growing, irregular lesion with multicolor pigmentation. Histologically, SSM presents with large pleomorphic epithelioid melanocytes showing nested groups of cells and single cells migrating intra-epidermally and/or in the superficial dermis. In contrast, typical for NM is a rapidly expanding nodule that may show ulceration and hemorrhage. Histologically, NM localize in the dermal component, likely combined to epidermal extension. LMM usually appears in chronically sun-exposed areas as a large, multicolor macule with irregular edges. The histological features include proliferation of atypical melanocytes, epidermal atrophy with the loss of rete ridges, severe dermal solar elastosis and dermal thinning. ALM occurs in the palms, soles and fingernail regions showing slowly growing macules like in LMM. Histologically ALM shows single atypical melanocytes scattered along the junctional epidermal layer (33).

### Melanoma-Associated Mutations

Melanoma is associated with a broad range of somatic mutations (34). The basal mutation frequency in melanoma is one of the highest of all cancer types (100/Mb entire exome) and most of them are point mutations of C → T (7). The most common mutation, found in around 50% of all melanoma cases, is the *BRAF* gene mutation, most often the *BRAF^V600E^* where valine is substituted with a glutamic acid (35). Interestingly, the *BRAF^V600E^* mutation can also be found in over 80% of benign nevi (36, 37) and is thought to be the driver mutation for the formation of acquired nevi (38). Other common mutations found in melanoma are *NRAS* (neuroblastoma RAS viral oncogene homolog), *c-Kit* (35) and *CDKN2A* (cyclin dependent kinase inhibitor 2A) the latter of which associates with familial atypical multiple mole melanoma syndrome (FAMMM) (39). Pigmentation and melanocyte differentiation related mutations in *MC1R* (melanocortin 1 receptor) and *MITF* (microphthalmia associated transcription factor) have also been found in melanoma. *MITF* regulates melanocyte proliferation, differentiation, and survival (40) and acts also as an oncogene regulating the functions of the tumor suppressor p16 and CDK2 (cyclin dependent kinase 2) (41, 42).

Other mutations occurring in melanoma are in genes encoding *CDK4* (cyclin-dependent kinase 4), *PTEN* (phosphatase and tensin homolog), *BAP1* (BRCA1 associated protein), *p53* and those associated with proper telomere function, such as *TERT* (telomerase reverse transcriptase) (39, 40, 43). In UV-related melanoma evolution the primary events depend on the age of the individual and the biological demeanor of the oncogenic initiating mutation in the nevi, such as BRAF. Then, the secondary events such as chronic irritation or irregular high dose UV exposure of mutation-bearing melanocytes damage the cell-cycle checkpoint regulatory system (G1/S phase) or telomerase function and result in even higher mutation burden and formation of melanoma cells (44, 45).

The aforementioned mutations often lead to the over-activation of signaling cascades involving MAPK (mitogen-activated protein kinase) and PI3K (phosphoinositol-3-kinase) pathways. Interestingly, these pathways activated in melanoma, act also closely in the regulation of hyaluronan synthesis (reviewed by Heldin et al. (2019) (46). The MAPK pathway (RAS/RAF/MEK/ERK) transduces extracellular signals from growth factors and hormones to proliferative responses, differentiation, and survival. PI3K(/AKT) signaling pathway is associated with cellular homeostasis. Loss of tumor suppressor gene *PTEN*, which acts as an inhibitor of AKT signaling, further increases the activation of PI3K signaling. The MAPK and PI3K pathways overlap in melanoma and therefore some signaling mediators such as NRAS act through both pathways. This complicates single-agent therapies since the other pathway can take over. Therefore, dual targeting is often needed (47, 48).

### Melanomagenesis

**Figure 2:**
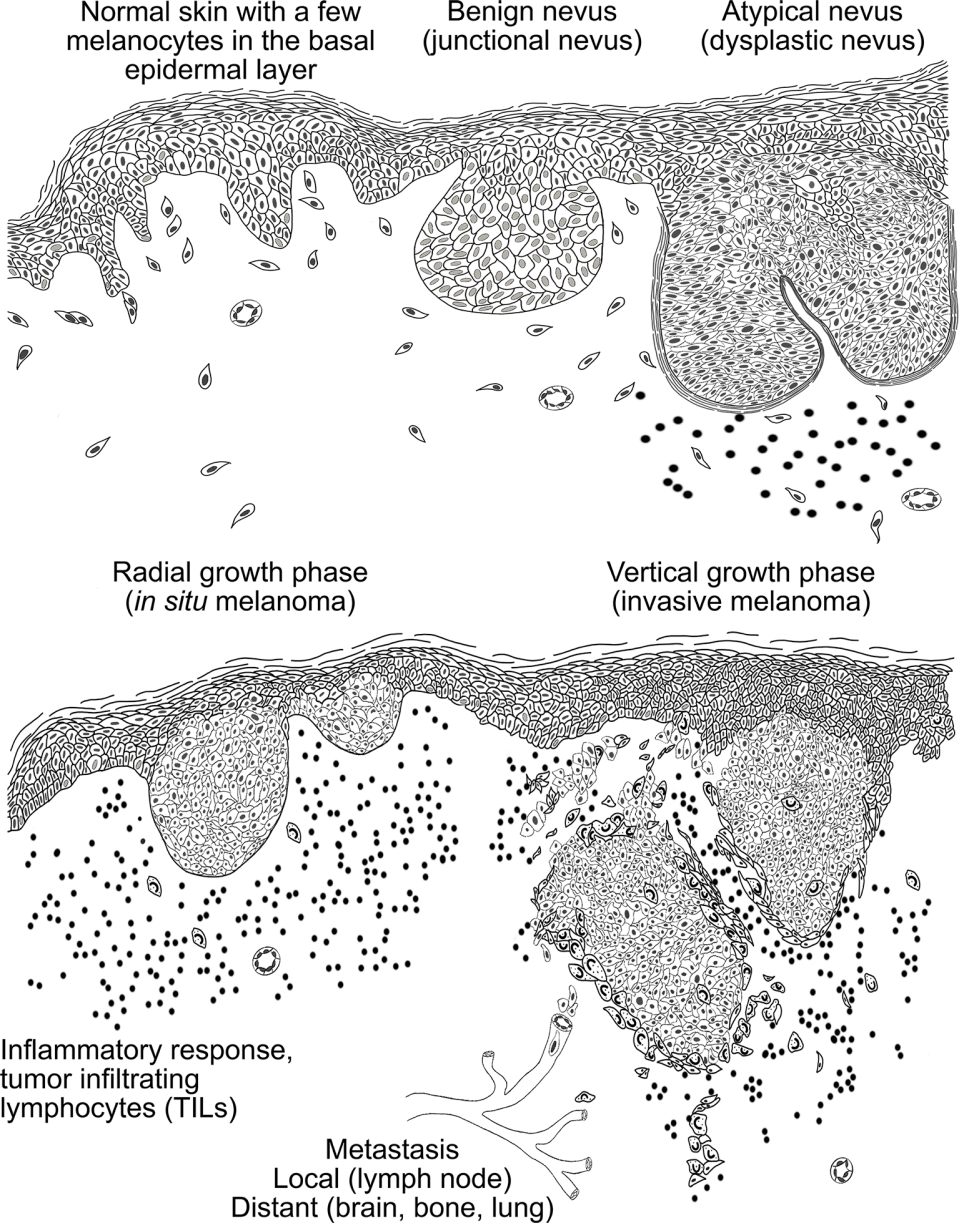
A schematic presentation of the histopathogenesis of the melanocytic tumors. In normal skin, melanocytes are scattered along the basal cell layer. Melanocytes accumulation leads to the formation of benign nevi that can reside in the epidermis (junctional nevus), in the dermis (intradermal nevus) or nevus cells can be assembled in both (compound nevus). In dysplastic nevus melanocytes exhibit cell hyperplasia and show cytological and architectural atypia. Moreover, lymphocyte infiltration around the nevus is typical in dysplastic nevi. In the radial growth phase (RGP) melanoma is still restricted to the epidermis by basement membrane and associates with high lymphocyte infiltration. When melanoma cells start to invade through the basement membrane into the dermis, melanoma has reached the vertical growth phase (VGP). In this stage the melanoma cells are capable of metastasizing *via* blood and lymphatic capillaries to local lymph nodes and distant organs.

Melanomagenesis (**Figure 2**) is a multistep process. Clustering of melanocytes forms a benign nevus without abnormal characteristics. These benign nevi can locate either in the epidermis (*junctional nevus*), or in dermis (*intradermal nevus*), or the nevus cell nests can be accumulated in both the epidermis and dermis (*compound nevus*). Melanocytic neoplasms arise from gain-of-function mutations in one or several primary oncogenes. The neoplastic changes usually occur as new lesions with dysplastic features in skin areas which have no previous melanocytic lesions (31). Dysplastic nevi embody cell hyperplasia, cytologic atypia and enlarged nuclei. They can remain in this regressed state for a long time or develop into radial growth phase (RGP) of melanoma. In this state, melanoma is still restricted to the epidermis by basement membrane and surgical excision is the preferred treatment of choice. The prognosis is good and the ten-year survival rate is over 90% (20, 49). When melanoma progresses from this step to the vertical growth phase (VGP) where melanoma cells invade through the basement membrane into dermis and are capable of metastasizing (locally or distantly), it has become the most aggressive type of skin cancer. At this point, the prognosis is inversely comparative to the depth of the neoplasm and the extent of lymph node metastases (50). The ten-year survival rates in stage III (lymph node positive) and deep (>4 mm) melanomas’ have improved during recent years due to novel therapies, but the prognosis largely depends on the sex and age of the patient, the anatomical location of the tumor, and the stage of melanoma at the time of diagnosis (51).

### Factors Affecting Melanomagenesis

One important function of UV is the induction of vitamin D precursor synthesis in the skin. For this purpose, the UV requirement is achieved already with the suberythematogenic doses (52). It has been estimated that 65% of all melanomas are caused by UV radiation. Excessive sun exposure during childhood is an important risk factor, but sunburns at any age increase the risk, as well as occupational exposure to UV for people who work outdoors (45). Acute UV exposure increases skin inflammation and DNA damages whereas chronic exposure induces immune cell infiltration and immune suppression leading eventually to malignant changes in melanocytes (26, 53). Within hours after UV exposure repair mechanisms start repairing the oxidative DNA damages (DNA single-strand breaks and DNA-protein crosslinks), and later on the cyclobutane pyrimidine dimers (CPDs). The CPDs are termed as UV signature mutations which induce C → T and CC → TT transitions mostly in the tumor suppressor p53 coding sequence (54). Reactive oxygen species (ROS) such as singlet oxygen, superoxide anion and hydrogen peroxide generated by chromophores, which absorb UVA- and UVB-photons, can also induce damages on nucleic acids, lipids or proteins (55). In addition to the DNA repair mechanisms, UV-induced damages launch multiple other repair mechanisms, such as secretion of paracrine factors (e.g. cytokines, chemokines and growth factors) by skin cells, activation of cellular protection mechanisms (e.g. cell-cycle checkpoints) and activation of cell death pathways and immune responses in damaged cells. At the cellular level, the most important function is the activation of tumor suppressor responses of p53 and p16 signaling pathways (45).

## Hyaluronan

### Hyaluronan and Its Synthesis

Hyaluronan is a peculiar polysaccharide with broad functions. Hyaluronan is involved in many physiological processes such as embryogenesis (56), wound healing (57), ECM organization (58–60), lubricant in the articular cartilage and synovial fluid (61, 62), mammary gland morphogenesis (63) etc. Hyaluronan, composed of cytosolic UDP-*N*-acetylglucosamine (UDP-GlcNAc) and UDP-glucuronic acid (UDP-GlcUA) precursors, is produced as a linear chain at the plasma membrane by hyaluronan synthases (HAS1-3), which are glycosyltransferases (64, 65). Hyaluronan synthesis by HASes can start *de novo* as the synthases do not need a precursor for the initiation. They use self-primer UDP-GlcNAc and add the two sugar substrates by turns to the reducing end of the growing chain (64). HASes are membrane-spanning enzymes that hold the catalytic site at the inner side of the plasma membrane and synthesized hyaluronan is protruded through a pore-like structure to the cell surface (64, 66). It is suggested that HASes can synthesize hyaluronan also intracellularly (67, 68), but the general perception is that HASes are functional and synthesize hyaluronan only at the plasma membrane (69). In normal physiology hyaluronan is produced as HMW size and can be up to 40,000 sugars long (8 MDa) (64). Extracellular and pericellular hyaluronan can be internalized *via* receptor-mediated (like CD44-mediated endocytosis) endocytosis for hyaluronan turnover and catabolism (70). Hyaluronan is degraded to LMW form either on the plasma membrane or in lysosomes by hyaluronidases (HYALs) (71), ROS (72) or UV (73). Albeit the mechanism of UV-induced hyaluronan degradation is less known. Moreover, TMEM2 (transmembrane protein 2) and HYBID (hyaluronan binding protein involved in hyaluronan depolymerization, also known as CEMIP or KIIAA1199) are also able to depolymerize hyaluronan (74, 75). TMEM2 degrades extracellular hyaluronan in a Ca^2+^ -dependent manner into intermediate-sized fragments which are thereafter internalized and completely degraded in the lysosomes (76). The HA degradation products, LMW hyaluronan, hyaluronan fragments (<250 kDa) and oligosaccharides (<20 kDa) are involved in inflammation (77, 78), wound healing (79) angiogenesis (80, 81) and in general they associate with pathological conditions, such as asthma and lung infections including coronavirus disease (82, 83) and cancer (84).

The enzymatic activity of the synthases also affects the molecular weight of the produced hyaluronan. It has been proposed that HAS1 and HAS2 synthesize higher molecular weight hyaluronan compared to HAS3 (85). However, recent studies have pointed out that HASes could be able to control the hyaluronan product size and the synthesis rate *in vivo* by separate enzyme submechanisms. This was shown in targeted mutation studies to B-X_7_-B motifs in *Streptococcus eguisimilis* HAS, which indicated that the different regions in the HAS enzyme are involved in hyaluronan size control as well as its enzymatic activity (86).

### Hyaluronan in Cancer

Understanding of hyaluronan in the inflammatory diseases and cancer is growing. Depending on which tissue type the cancer originates from, the increased/decreased content of hyaluronan in the tumor or stromal cells correlates with tumor aggressivity, poor prognosis, and recurrence of the cancer. Breast cancer, which originates from simple type of epithelium that contains low levels of hyaluronan in the normal state, shows increased hyaluronan content in the tumor cells and in the stroma, and the amount of hyaluronan associates with aggressive type of breast cancer and poor overall survival (14, 17). The trend is similar in other cancers originating from simple epithelia. Increased content of hyaluronan in the stroma of ovarian cancer (87, 88), prostate cancer (15, 16, 89) and pancreatic cancers (90) indicates poor prognosis. Similar findings have been demonstrated in gastric cancer parenchyma (91) and in tumor cells (92) and tumor interstitial fluid of colorectal cancer (93).

**Figure 3:**
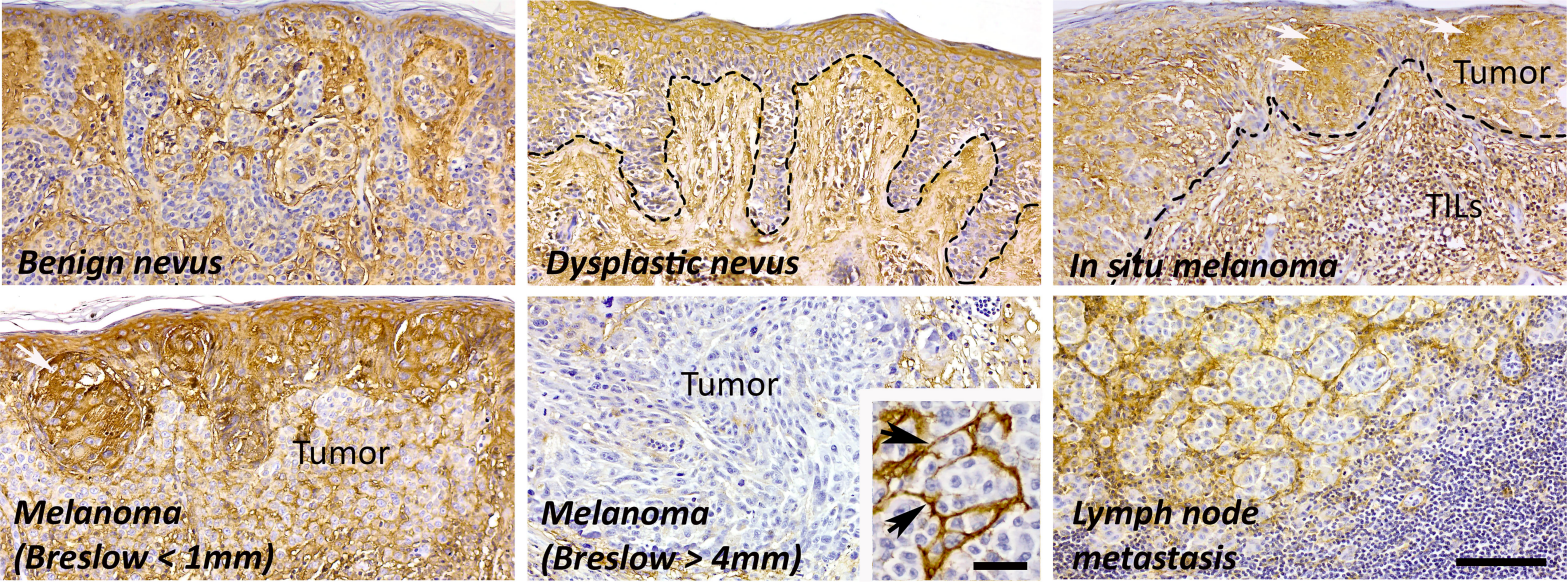
The amount of hyaluronan in different stages of melanoma. In normal skin, both epidermis and dermis are highly hyaluronan positive, also melanocytes in the benign nevi show moderate or intense hyaluronan staining. The amount of hyaluronan increases in dysplastic nevi and in local tumors (white arrows) probably as part of the inflammatory reaction with elevated number of tumor infiltrating lymphocytes (TILs). In invasive melanoma, the amount of hyaluronan is decreased in tumor tissue but the stroma is strongly hyaluronan positive (black arrows). The hyaluronan staining pattern is similar in lymph node metastases and in deep melanomas (Breslow > 4 mm). Scale bar 100 µm, 40 µm in insert.

In cutaneous melanoma, hyaluronan metabolism is different compared to cancers originating from simple epithelia. The hyaluronan content varies depending on the stage of the tumor (**Figure 3**). Cultured skin melanocytes contain high amount of pericellular hyaluronan (94) similarly to the cells in the benign nevi (18). Moreover, the stroma between the melanocytic nests in the benign nevi shows intense staining for hyaluronan. In *dysplastic nevus*, where melanocytic cells show cytological and architectural atypia, the content of hyaluronan is elevated to local melanomas (melanoma *in situ*), where tumor cells show intense membraneous and cytoplasmic staining for hyaluronan. When melanoma progresses and tumor cells start to invade vertically, tumoral hyaluronan content decreases (melanoma, Breslow < 1mm) while the stroma remains hyaluronan positive (18). Especially in nodular type of tumors the stroma shows intensive staining for hyaluronan (melanoma, Breslow > 4mm, *insert*). Hyaluronan staining pattern in lymph node metastases is similar to deep melanomas. The reduction of hyaluronan in the tumor tissue has been shown to be due to the loss of HAS1 and HAS2, and increased HYAL2, and this associated with poor prognosis and shortened disease-specific survival in melanoma (19, 95).

### The Effects of UVB on Hyaluronan Metabolism

Mouse studies have shown that chronic UV exposure with minimal erythemal dose induces the accumulation of hyaluronan in the epidermis, which correlates with epidermal hyperplasia (96). In organotypic keratinocyte cultures, UV-exposure was shown to increase hyaluronan metabolism by upregulating the expression of both HASes and HYALs simultaneously and by shifting the molecular weight of hyaluronan towards smaller size range (97). Studies with keratinocytes and dermal fibroblasts have shown different temporal expression patterns of HASes and HYALs after UVB-exposure (98). Immediate responses after UVB-exposure showed decrease in hyaluronan secretion in both cell types whereas 24 hours later keratinocytes showed increased hyaluronan synthesis while in dermal fibroblasts hyaluronan synthesis was still downmodulated. 24 hours after UVB-exposure the expression of *HAS1* and *HAS2* was upregulated in both cell types, and in addition the expression of *HYAL1* and *-2* was elevated in dermal fibroblasts indicating increased hyaluronan degradation after UVB exposure (98). The similar mechanism was also detected in photoexposed human skin, where LMW hyaluronan content was increased due to upregulated expression of HYALs compared to photoprotected skin (99). In addition to increased HYAL activity after UV exposure, UV induces oxidative stress *via* ROS production (100), which can further induce hyaluronan fragmentation (72, 101). Hyaluronan is able to scavenge toxic ROS (102, 103), or inhibit their formation, but at the same time hyaluronan is fragmented (103). Albeit it is not yet known how ROS-induced hyaluronan degradation and generation of LMW hyaluronan followed by inflammation in skin contributes to melanoma development in melanocytes, the research is supporting the idea that hyaluronan has a role in melanoma development and especially affecting the TME in melanoma (18, 19, 94, 104).

### Hyaluronan in Inflammatory Reactions

As mentioned earlier, UV radiation induces various biological effects in the skin such as DNA damage, pigmentation and inflammation. Excessive exposure to UVB causes sunburn reaction together with inflammation. Several cell types, such as keratinocytes, fibroblasts, Langerhans cells, melanocytes and macrophages, participate in these inflammatory reactions by secreting inflammatory mediators and reshaping the tissue structure. During inflammation, the amount of hyaluronan is elevated (105, 106), and especially the LMW hyaluronan and hyaluronan fragments are associated with inflammation (107). They can activate intracellular signaling cascades and induce cytokine and chemokine production *via* receptor activation. LMW hyaluronan, for example, has shown to induce the production of IL-6, IL-8, CXCL1, CXCL2 and CXCL6 in dermal fibroblasts (108). Key receptors for LMW hyaluronan include CD44 (cluster of differentiation 44), RHAMM (receptor for hyaluronan-mediated motility), TLR2 (Toll-like receptor) and TLR4 (109). TLR4 activation leads to NF-κB-mediated signaling and production of inflammatory cytokines and chemokines such as IL-6, IL-8, CXCL-1 and CXCL-10 in melanocytes (94) and IL-8 and MMP-2 secretion in melanoma cells (110). Hyaluronan fragments of 4-16 oligosaccharides, in turn, have shown to induce the maturation of dendritic cells by autocrine production of TNF-α (81), and dendritic cell-associated hyaluronan appears to contribute to the antigen-specific activation of T-cells (111). We have recently shown in primary melanocytes that UVB-exposure mediated hyaluronan coat degradation promoted the expression of proinflammatory cytokines IL-6 and chemokines (IL-8, CXCL1, CXCL10) *via* TLR4 (94). Interestingly, in this model, the expression of proinflammatory cytokines and chemokines were suppressed by *HAS2* silencing, suggesting that HAS2 expression and hyaluronan fragments support the formation of pro-inflammatory state. In contrast, HMW hyaluronan (970 kDa) has been shown to protect epidermal keratinocytes against UVB-mediated TGF-β, IL-6 and IL-8 production (112). It is hypothesized that HMW hyaluronan has a protective role in skin against UV radiation and the fragmentation of hyaluronan acts as a damage-associated molecular pattern (DAMP) (113).

The effects of hyaluronan on immune, non-malignant and malignant cells are size-dependent. HMW hyaluronan has been shown to be relatively inert, whereas its degradation products have been speculated to be biologically active and modulate the TME towards pro- or anti-inflammatory states depending on the tumor stage. Hyaluronan and its fragments are thought to regulate macrophage polarization, although the exact mechanisms are still unresolved (114). Macrophages have an essential role in orchestrating the inflammatory responses and they are also a key component of TME in all stages of melanoma development (115, 116). In the TME, tumor associated macrophages (TAMs) are very plastic and are composed of several distinct populations that share features of both pro-inflammatory (M1) and immunosuppressive (M2) macrophages (117). Intermediate-size hyaluronan (50-1000 kDa) fragments have shown to activate monocyte polarization to immunosuppressive M2-type macrophages *via* the activation of TLR4 (118), but there are also contrasting studies showing that hyaluronan fragments induce M1 polarization of macrophages *via* TLR4 (119). In addition, TLR4 signaling pathway is responsible for inflammatory gene expression induced by hyaluronan fragments in several other cell types like dendritic cells, melanocytes and tumor cells (81, 94, 110, 119, 120).

In TME, enhanced hyaluronan production by stromal fibroblasts (104, 121) combined to increased hyaluronidase activity from melanoma cells could produce broad pool of different types of hyaluronan fragments and oligosaccharides that maintain the inflammatory milieu. This is also supported by a study from Sapudom et al. (2020) where hyaluronidase added to decellularized fibroblast matrix, 3D fibroblast matrix and hyaluronan-functionalized 3D collagen matrix increased melanoma cell proliferation and invasion due to lower hyaluronan molecular size (122). It has also been shown that LMW hyaluronan promotes melanoma lymph node metastasis (123), soluble LMW hyaluronan contributes to melanoma cell proliferation (124), and shorter hyaluronan fragments induce TLR4-mediated signaling in melanoma and promote inflammation as well as invasiveness (110). Interestingly, in naked mole rats HAS2 produces exceptionally high molecular weight hyaluronan that is able to avoid fragmentation (125). These animals display longevity and are cancer-resistant, but when HAS2 is knocked down or HYAL2 is overexpressed the naked mole rat cells became prone to malignant transformation (125).

Current understanding suggests that hyaluronan fragments modify the adaptive immune responsiveness and shape the microenvironment (113). The formation of cancer inflammatory milieu recruits immune cells to the TME to shape it suitable for tumor progression. TLR4 has shown to be overexpressed in melanoma tumors and associate to decreased survival (126), which links hyaluronan and TLR4 in melanomagenesis. TLRs respond to acute inflammatory signals either from microbial sources (PAMPs, pathogen-associated molecular patterns) or self-derived stress signals (DAMPs), the role that also hyaluronan has been proposed to play. The activation of TLR signaling leads to NF-κB, AP-1 and/or IRF-3 mediated inflammatory response in healthy skin and in primary melanocytes and eventually skin carcinogenesis (94, 127). This suggests a role for hyaluronan metabolism, especially for UV-induced hyaluronan fragmentation *via* TLR4-mediated inflammation, in melanocyte transformation process towards cutaneous melanoma. Future research should aim at clarifying whether chronic UV-exposure together with hyaluronan fragmentation could mediate sustained inflammatory responses initiating cutaneous melanoma development. Even though hyaluronan can modulate immune responses, it may also act as a protective barrier in the TME and thus prevent immune cells to reach and recognize the tumor cells (128). And in this way hyaluronan may also promote the immune evasion of tumor cells.

## Melanoma Tumor Microenvironment

### Composition of Different Cell Types in the Tumor Microenvironment

TME is inherently diverse surroundings consisting of not only the tumor and stromal cells but also immune cells, blood vessels and the ECM, which all affect the tumor progression. Considering the complexity of the TME, cancer therapies cannot focus only on the tumor cells (129, 130). Melanoma is one of the most immunogenic cancers and immune cells have a significant role in all stages of tumor progression, from the initiation to the metastatic processes and thus these cells are the main target of modern anti-melanoma therapy. Transformed melanocytic cells start to express tumor-specific antigens (TSA), which induce the infiltration of dendritic cells, which later activate the cells of innate and adaptive immunity. Dendritic cell maturation is needed to actuate the T-cell mediated anti-tumor response and natural killer cell activation (131). Lymphocytes infiltrate the tumor environment in response to chronic inflammation. These so-called tumor-infiltrating lymphocytes (TILs) appear to be a positive indicator for melanoma prognosis (132, 133). TILs are a heterogeneous population of lymphatic cells such as T-cells, natural killer cells and myeloid-derived suppressor cells (134). The presence of TILs promotes immune responses against tumor cells and can be thought as a predictive biomarker in melanoma. Computational meta-analysis of melanoma tumors indicated a favorable prognostic role for CD3+, CD4+, CD8+, FOXP3+ and CD20+ positive TILs in the overall survival when the amount of TILs in the tumor was high (135). A study by Erdag et al. (2012) categorized tumor microenvironments of metastatic melanoma into three immunotype subgroups A, B and C based on the number and localization of TILs (T-cells, B-cells, natural killer cells), mature dendritic cells and macrophages. The immunotype A had no immune cell infiltration and the median estimate of survival in this group was only 15 months. The immunotype B had infiltration of immune cells to regions proximal to intratumoral blood vessels with the median estimate of survival was 23 months. The last immunotype C had immune cell infiltration throughout the metastatic tumor with a median estimate of survival of 130 months (136). Although the number of TILs in melanoma as a prognostic biomarker, especially in the VGP, was established a long time ago (137), it is still a valid diagnostic predictor estimating patients’ survival.

In contrast to TILs, tumor-associated macrophages (TAMs) can promote the dissemination of cancer cells and expansion of the tumor depending on their type. The categorization of TAMs is not unambiguous. Traditionally, TAMs are categorized as classically activated macrophages (M1) that secrete pro-inflammatory cytokines and have anti-tumor effects, and alternatively activated macrophages (M2) that produce anti-inflammatory cytokines and have protumor effects (138). As already mentioned earlier, in the TME, TAMs are composed of several distinct populations that share features of both pro-inflammatory and immunosuppressive macrophages and these cells induce and maintain pro-tumor inflammation, which promotes tumor growth, invasion, angiogenesis, stemness and immune suppression (139, 140). The precise mechanisms underlying TAM-mediated immune suppression in TME are still unknown but at least partly relate to their ability to inhibit cytotoxic activity of CD8^+^ T cells *via* PD-1 (programmed cell death protein) (141). PD-1 is an immune checkpoint receptor that prevents overstimulation of immune responses upon engagement to its ligand, PD-L1 (142). In several cancers, including melanoma, the expression of PD-L1 is considered as a marker for immunosuppressive TME (143). During tumor progression, TAMs start to overexpress PD-L1, like tumor cells, and thus are able to downmodulate the function of CD8+ lymphocytes and facilitate the immune evasion of tumor cells (144). Moreover, it has been shown *in vitro* that alternatively activated macrophages can suppress T-cell function by producing regulatory cytokines, such as TGFβ and IL-10, hence repress T-cell proliferation (145) and recruit regulatory T-cells to the immune suppressed TME, which further contributes to tumor progression (138).

Salmi et al. (2019) reported that the number of TAMs is higher in malignant melanocytic lesions compared to benign nevi and TAMs are localized especially in the tumor nests in deep melanomas. They also reported that the dysplastic nevi have higher number of CD163+ macrophages (M2 type macrophages) compared to benign nevi, which could predict immunosuppression and progression of melanoma (146). It is also known that these benign and dysplastic nevi as well as melanoma *in situ* express hyaluronan abundantly (18), but its role for macrophage polarization is still unknown. It has been shown that HMW hyaluronan suppresses M1 macrophage polarization and promotes the immunosuppressive activation in induced pulmonary inflammation (147). Kim et al. (2019) also showed *in vitro* that hyaluronan in the ECM induces M2-type polarization of THP-1 cells (148). The similar mechanism could also be involved in the formation of immunosuppressive microenvironment in melanoma, where increased hyaluronan synthesis at the early stage of melanoma could polarize macrophages toward immunosuppressive phenotype. Although it is generally thought that M2 type TAMs have protumor effects in the TME, the pro-inflammatory macrophages have also shown to secrete several protumor factors, like TNFα and IL1β, and are able to modify the TME suitable for cancer progression (149–151).

In addition to several immune related cells, TME contains fibroblasts which are mainly responsible for the production of complex extracellular matrix, which either suppresses or supports the tumor progression. In the early stages, in pro-inflammatory tumors, factors like IL-6, TNFα, PDGF and ROS secreted from tumor and immune cells can activate the stromal fibroblasts. Once activated these contractile and highly elongated cancer associated fibroblasts (CAFs) produce several cytokines and chemokines, synthesize and degrade matrix components and generate tensile forces, which all are needed for tissue remodeling for tumor cell growth and spreading (152).

### Extracellular Matrix in Melanoma

The tumor ECM is a mixed territory of different types of proteins, glycoproteins, proteoglycans and glycosaminoglycans, which all have specific physiological and biochemical properties (153). The composition of ECM maintains the three-dimensional structure that keeps up tissue homeostasis, undergoes continuous remodeling and contains over 100 different proteins and bioactive molecules like growth factors, cytokines and extracellular vesicles (EVs) produced jointly by stromal cells and tumor cells (154). A typical ECM matrix in melanoma includes fibrillar and structural proteins, such as collagen types of I, III, VII, XV, XVIII, laminin 7, tenascin-C, fibronectin and hydrated gel-forming macromolecules like hyaluronan and proteoglycans, as well as integrins, which carry out the adhesive signaling (155). Excess of these ECM components, such as collagen and fibronectin, increase tissue fibrosis and hence matrix stiffness, which affects the metastatic potential of tumor cells and invasiveness (156). Li et al. (2019) showed in melanoma cells that increased matrix stiffness enhanced the metastatic potential of naturally less-metastatic melanoma cells. Their metastatic potential was comparable to melanoma cells with high metastatic potential in stiffer matrix. This indicates that the tumor-surrounding matrix has a high impact on oncogenesis and changes in the matrix might have greater effect on the metastatic process than tumor cells (157).

A rapidly growing cell mass causes dysfunctional tumor vasculature leading to hypoxic environment and low oxygen levels. Hypoxia can lead to specific changes in signaling pathways, such as ERK hyperactivation leading to increased invasiveness, angiogenesis, EMT changes, radiotherapy resistance, changes in the ECM composition, which all promote tumor progression. HIF (hypoxia-inducible factor) 1 and 2 are regulators of hypoxia-mediated responses in over 300 target genes. Hypoxic TME correlates with aggressive cancer phenotype associated with increased tumor plasticity and may induce cancer stem-like cells (158–160). The high metabolic activity of melanoma cells causes the production of acidic molecules to the TME, which affects the ECM composition. Acidification of the TME influences the tumor cell invasiveness, increases expression of EMT-related proteins and causes a selection of acid-resistant cells that show greater metastatic potential. Acidic TME also affects anticancer therapies and the absorption of mildly alkaline chemotherapeutics leading to melanoma cells drug resistance (159).

The composition of the ECM changes during tumor development. Many ECM molecules are expressed at low levels in benign nevi whereas their levels increase in metastatic melanoma, as shown with tenascin-C, fibronectin and different epitopes of heparan sulfate. Other glycosaminoglycans such as chondroitin sulfate and hyaluronan are highly expressed in the benign nevi and also abundant in melanomas either in the tumor nests or in the stroma (18, 161, 162). Recently, Mayorca-Guiliani et al. (2017) developed an ISDoT (*in situ* decellularization of tissues) technique where they could study the native tumor ECM in a mouse model. They could identify more than 200 ECM proteins as well as many secreted factors such as MMPs, versican and members of the S100 protein family from the decellularized tumor tissues, showing tumor-specific changes in the ECM composition in comparison to healthy tissue (163).

Phenotype switch is an important regulator between proliferative and invasive states of melanoma cells. Melanoma cells do not undergo normal EMT changes, however they utilize EMT and MET (mesenchymal-to-epithelial transition) states that are mediated by adhesion molecules such as Cadherin-11, Connexis, integrins, N-cadherin and MCAM (CD146) which all are indicators of progressed melanoma (164). Melanoma cell phenotype switch can also alter MITF expression, which in melanocytes regulates pigmentation. High MITF expression sensitizes melanoma cells to BRAF and MEK inhibitors whereas the cells are more proliferative. However, low MITF expression increases drug resistance and induces invasive phenotype. Low expression or loss of MITF was also shown to increase glycosaminoglycan metabolism, ECM organization and structural organization in melanoma cells. Also, loss of MITF increased stem cell marker SOX2 that is important in melanoma cell self-renewal. Thereby, the level of MITF alters melanoma phenotype switch as well as matrix composition and interactions (164, 165).

Ubiquitous hyaluronan in the ECM provides the cells a suitable environment to migrate, divide, change their shape and adhere due to its viscous hygroscopic nature. In addition, hyaluronan facilitates signaling from the environment, but at the same time also orchestrates many unwanted functions in the ECM by permitting the cancer cells do the same (66, 166). Even though the role of hyaluronan is broadly studied in the context of melanoma cells *in vitro*, its function in primary melanocytes and early melanoma development is uncertain. Neither is known how the basal level of hyaluronan differs in different skin types or the alteration of hyaluronan in photoprotected areas versus photoexposed areas, as UV exposure has been shown to increase the hyaluronan content in epidermis (99). It is thought that hyaluronan in the ECM could act as a protective matrix and hinder the inflammatory reaction produced by ROS (10). The thicker hyaluronan content in the skin could shield from the harmful effects of UV, reduce the inflammatory reaction following it, and therefore protect the skin cells against carcinogenesis. When hyaluronan degradation was combined to UV-exposure in primary melanocytes, the inflammatory reaction following it was highly enhanced (94). This could indicate that thicker hyaluronan coat around the cells and in the ECM could moderate the inflammatory reaction following UV exposure. UV-induced conditions also associate with hyaluronan degradation, which further enhances the inflammation. The direct mechanism of hyaluronan fragmentation by UV is still unknown, but the activation of HYAL and/or ROS directly, by ROS-activated HYAL or direct fragmentation by UV have been suggested. Inflammation following UV exposure is also an acute defense mechanism to protect and dispose damaged cells (167), but in a chronic state it can lead to carcinogenesis.

### Melanoma-Stroma Interaction

**Figure 4:**
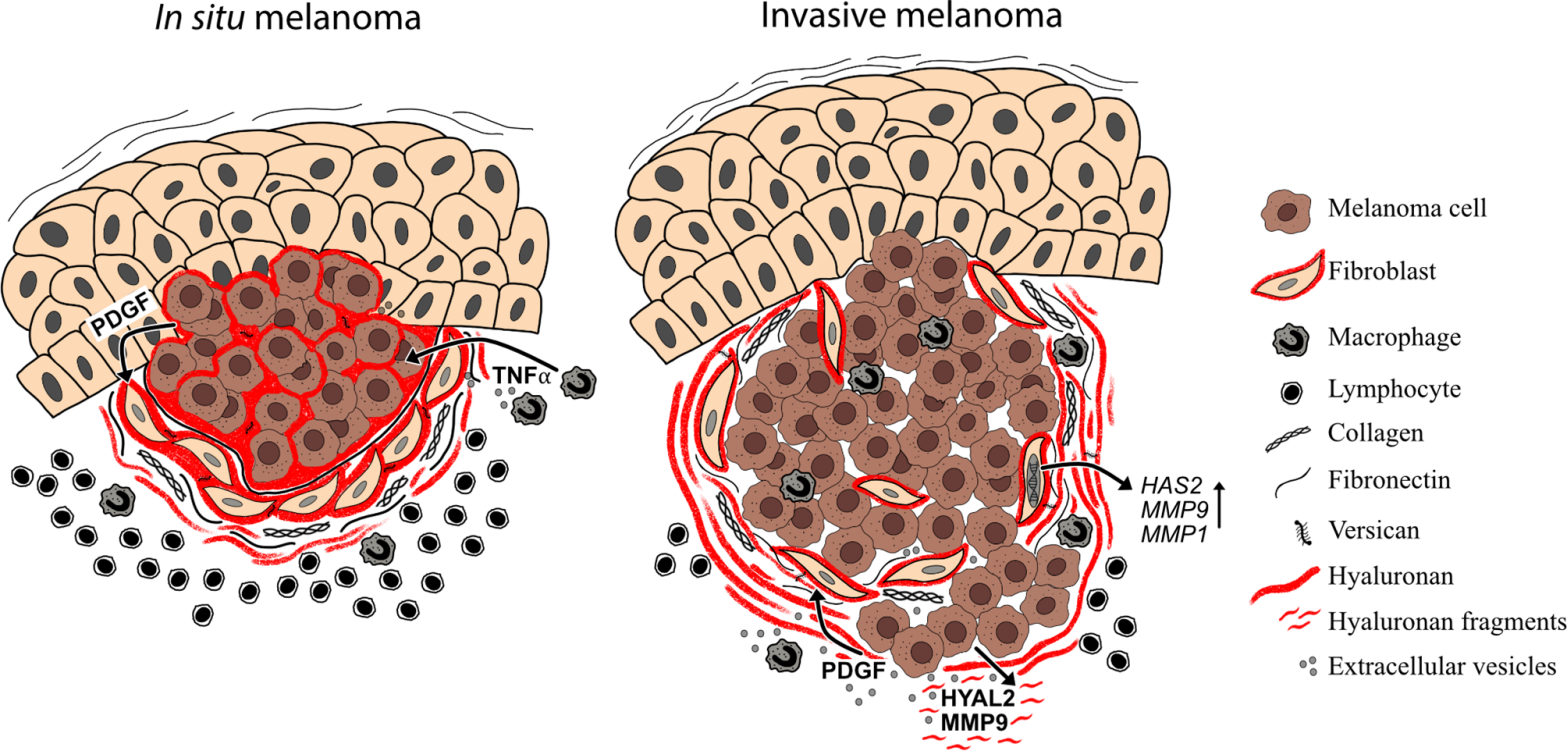
Melanoma-stroma-ECM in superficial and deep melanomas. Superficial melanomas (*in situ* melanoma) have high tumoral hyaluronan content and the stroma contains also abundantly hyaluronan, which is produced by cancer associated fibroblasts. In addition to hyaluronan, melanoma stroma contains other ECM components such as type I collagen, fibronectin and versican. This hyaluronan-rich pericellular matrix surrounding the melanoma cells may mask the tumor cells from immune cell recognition. In deep melanomas (i.e. invasive melanomas), the tumoral hyaluronan content is decreased but stroma is still hyaluronan positive. The hyaluronan and hyaluronan fragment -rich matrix in the tumor stroma may, in turn, facilitate the invasion and metastasis of melanoma cells. In the TME, soluble growth factors (e.g. PDGF), cytokines (e.g. TNFα) and extracellular vesicles, produced by both tumor and stromal cells regulate the expression of HAS2, HYAL2 and MMPs.

It is well known that the interaction between cancer cells and stroma is needed for tumor progression and metastasis. Already in 1889 Paget’s theory of “seed and soil” emerged (168). Since that, different studies on melanoma-tumor stroma have emerged and nowadays is well acknowledged that the surrounding tumor stroma is equally important as the tumor itself. Our results (2012) as well as others have shown that melanoma cell-secreted factors (PDGF) induce stromal fibroblasts to produce hyaluronan by upregulating *HAS2 via* PDGFR-PI3K-AKT and p38 MAPK signaling (104, 121). In this study, PDGF -induced *HAS2* upregulation associated with activated phenotype of fibroblasts with elongated morphology, upregulation of proteolytic enzyme MMP1, MMP9 and MT1-MMP expressions and increased invasion into the matrix, suggesting that HAS2 is essential in activating the fibroblasts in the tumor stroma (104). The activated fibroblasts, so called cancer associated fibroblasts (CAFs), facilitate melanoma cell invasion by modifying the ECM suitable for tumor invasion (169). These *in vitro* results are in line with the patient data where Siiskonen et al. (2013) showed that hyaluronan staining in invasive and metastatic melanoma stroma is intense in comparison to almost negative tumor staining. They also showed that HYAL2 staining is elevated in invasive and metastatic melanoma samples, which could indicate that hyaluronan catabolism and thus the degradation of hyaluronan is increased in melanoma cells (18) (**Figure 4**). Increased hyaluronidase activity in melanoma cells has also been shown to induce angiogenesis (170) and cutaneous wound healing (171) likely attributed to hyaluronan fragments. As it has been shown that tumor cells secrete higher amounts of extracellular vesicles (exosomes, microvesicles) compared to non-transformed cells (172), their role in tumor-stroma interaction is essential. Recently Arasu et al. (2020) published a study where they reported that HAS3-induced hyaluronan coated extracellular vesicles (EV’s) from melanoma cells induced EMT changes and increased proliferation of target cells, keratinocytes, similarly as CAF -derived hyaluronan has shown to promote melanoma cell proliferation (121, 173). In Arasu’s study they hypothesized that hyaluronan coated EV’s may form a positive feedback regulation which induces the tumorigenic potential of the target cells in the surroundings (173). This EV-mediated target cell activation has also been shown to be bidirectional. Cancer associated fibroblasts’ (CAFs) exosome secretion was enhanced by melanoma cell-secreted exosomal miRNA. These CAF-derived exosomes promoted melanoma progression and metastasis (174). In addition, EV’s can carry matrix-remodeling proteins, such as MMPs, TIMPs, ADAMTSs, hyaluronidases, heparanases, whose proteolytic activity can modify the composition of the ECM suitable for tumor progression (175).

### Therapeutic Approaches in Melanoma

The therapeutic approaches in melanoma depend on the prognostic stage of the disease. The 8^th^ American Joint Committee on Cancer staging and classification system (AJCC8) is the recommended standard for melanoma staging (176). The standard of care for local or locally advanced melanoma (AJCC8 stage I-III) is radical surgical excision with either sentinel lymph node biopsy or regional lymph node evacuation depending on the clinical preoperative lymph node status (176–179). Although surgical removal of oligometastases might be an option for selected patients with stage IV melanomas, the backbone of the treatment of metastatic melanoma is systemic oncological therapy with immunomodulating agents (immune check point inhibitors, ICIs) and/or BRAF-signaling route-targeted therapy. During recent years, these agents have also been available for the adjuvant treatment of high-risk stage IIIB-D and IV diseases, where the 5-year melanoma-specific survival is 85% or less (176, 180–185).

Considering the high immunogenicity of melanoma it is not surprising that the therapeutic options targeting the immune cell-TME signaling have turned out effective. Immune checkpoint inhibitors (nivolumab or pembrolizumab, PD-1 antibodies) enhance the anti-tumor immune response by blocking the signaling *via* suppressive immune checkpoint molecules PD-1, PD-L1 and CTLA-4 that are often expressed in the TME by tumor cells and immune cells, such as tumor associated macrophages (TAMs), as a mechanism of immune evasion. At best, immune checkpoint inhibitors can induce complete and durable tumor regression of metastatic diseases (186, 187). However, therapy resistance is common and severe, even fatal, autoimmune-mediated toxicities are associated with the therapy (186–188). Biomarkers predictive of the therapy result are therefore needed to estimate the response likelihood and to optimize the patient selection for therapy. So far, the only clinically used predictive biomarker of the efficacy for immune checkpoint inhibitor therapy, is the expression intensity of PD-L1 in the TME, but it has turned out to be suboptimal (188, 189). Other potential biomarkers are under investigation. These include the TME lymphocyte infiltration (190, 191) and tumor mutation burden (192). Moreover, as the structure of ECM in melanoma stroma may affect the therapy response, its potential as one of the predictive parameters should also be evaluated.

Hundreds of ongoing clinical trial studies are currently exploring other immunotherapeutic targets for future melanoma treatment (ClinicalTrials.org). The furthest developed novel anti-check point molecule, with recently released phase II/III results, is the LAG-3-inhibitor relatlimab, which has shown clinical efficacy in combination with nivolumab in metastatic melanoma (193). The interleukin 2 pathway agonist bempegaldesleukin, with the currently ongoing phase III clinical trial, and has shown promising phase II results in combination with nivolumab. An IL-6 antagonist, tocilizumab, is also studied in combination with nivolumab in a phase II study (Clinicaltrials.gov, NCT03999749).

A local treatment option for unresectable stage IIIB-IVM1a melanomas with (sub)cutaneous metastases is an oncolytic vaccine called talimogene laherparepvec (T-VEC; IMLYGIC^®^), a modified herpes virus vector that is injected locally in melanoma lesions and, in addition to direct cell destruction, enhances both local and distant immune responses. The use of T-VEC in advanced melanoma is also being evaluated in combination therapy approaches, e.g. together with ipilimumab and pembrolizumab (194, 195), with the aim of increased efficacy in patients with visceral metastases. Other vaccine therapies, including dendritic cell-based, peptide-based and vector-based vaccines are also in the testing phase and so far, the results have not been assuring (196). Instead, adoptive cell therapies (ACT) are a promising investigational option. The ACT approaches include tumor infiltrating lymphocytes (TILs) and chimeric antigen receptor (CAR) T-cell therapy (197).

## Therapeutically Targeting Hyaluronan in MELANOMA

Therapeutic targeting of hyaluronan degradation or combining it with other types of therapy have been studied in solid tumors associating with high accumulation of hyaluronan in the tumor or in the stroma like in pancreatic cancer (198). *In vitro* studies have shown that hyaluronan-rich matrix constrains the access of immune cells, like CD8+ cytotoxic T cells, to the tumor area in tumors with high hyaluronan matrix such as breast and pancreatic cancer (199). Using a PEGylated recombinant human PH20 hyaluronidase (PEGPH20) in combination with trastuzumab or cetuximab, the immune cell infiltration was enhanced but also the used treatment, trastuzumab- or cetuximab-mediated antibody-dependent cell-mediated cytotoxicity against tumor cells was highly improved. Similar results were seen in mouse models (200). However, a phase I trial in pancreatic ductal adenocarcinoma patients using PEGPH20 in combination with gemcitabine resulted in unacceptable adverse effects such as musculoskeletal pain, peripheral edema and thromboembolic events (201). Moreover, the overall survival or the progression-free survival were not improved in phase III trials of pancreatic ductal adenocarcinoma with the treatment of PEGPH20 in combination neither with nab-paclitaxel nor gemcitabine. Patients also suffered higher grade adverse effects such as fatigue, muscle spasms and hyponatreamia (202). Nevertheless, in 2020, the FDA approved another type of hyaluronidase, hyaluronidase-zzxf from Phesco, to be used as a subcutaneous injection to increase dispersion and absorption of pertuzumab or trastuzumab to treat HER2-positive early stage and metastatic breast cancer (203).

Since the hyaluronan levels in advanced stage melanoma tumors are low or almost negative (18) in contrast to breast and pancreatic cancers, similar kind of hyaluronidase therapies most likely cannot be used. However, *in vitro* studies have shown that melanoma cells induce hyaluronan production in dermal fibroblasts (104, 121) and it also correlates with the hyaluronan levels in the stroma of melanoma (18), and this could be a potential target for therapy. Theoretically, targeting the stromal hyaluronan in melanoma might induce better immune cell recognition and T-cell infiltration, as was seen in pancreatic ductal adenocarcinoma (204). Bearing this in mind, new local photothermal therapies (PTT) in synergy with immune therapy have been tested by combining dissolved microneedles to hyaluronidase-modified semiconductor polymer nanoparticles containing poly(cyclopentadithiophene-*alt*-benzothiazole) and immune adjuvant polyinosinic-polycytidylic acid in mouse models. The approach enhanced the activation of immune cells, especially T-cell-mediated immune responses. Although, its use is currently limited only to superficial melanomas as a local treatment (205), it opens prospects to combine targeted hyaluronan degradation and immunotherapy to treat melanoma TME in the future. In addition, 4-methylumbelliferone (4-MU) is an interesting agent known to inhibit hyaluronan synthesis (206). 4-MU acts as a competitive substrate for UDP-glucuronosyltransferase -enzyme involved in hyaluronan synthesis (207), it inhibits hyaluronan synthesis by depleting its precursor sugar UDP-glucuronic acid and downregulating the expression of HAS2 and HAS3 (208). *In vitro*, 4-MU has shown to inhibit melanoma cell proliferation and invasion into collagen lattices (209). Recently, a topically applied 4-MU formulation was shown to decrease cutaneous hyaluronan content in a mouse model (210), opening interesting new approaches to treatment of conditions with altered hyaluronan metabolism, including melanoma.

## Conclusions and Future Perspectives

Considering the yearly increasing numbers of new melanoma cases, the race in finding effective therapies remains tense. Preventive measures seem to be cost-effective (211) but they may not reach people at the greatest risk of developing skin cancer. Luckily, basic clinical research has taken huge steps during the past years and decades and enlightened the details of melanoma progression. Not only has our understanding of the several genes and signal transmitters involved in the pathogenesis of melanoma increased enormously, but we are also starting to realize the complexity of the microenvironment surrounding the evolving tumor. As reviewed here, hyaluronan, an abundant glycosaminoglycan surrounding skin cells, has also several functions affecting the development of melanoma. Hyaluronan may have controversial role in cancer as it is increased in certain cancer types but decreased in others. Although there are effective treatments available and promising therapies being studied in clinical trials, we still need a more detailed understanding of the complexity of the ECM, including hyaluronan, in melanoma progression. Future research should focus on investigating the gradual processes in the TME during tumor development and on finding new ways to modulate the matrix surrounding the tumor. The key to conquer cancer may not be just affecting the tumor cells or the immune reactions but also to modify the TME susceptible for treatment. Hyaluronan appears to be a potent molecular target for future therapeutic modalities and innovative research ideas should be supported. Considering the past advances in cancer biology, the breakthrough findings and the most effective treatments are yet to come.

## Author Contributions

Writing - Original Draft Preparation: PT. Writing - Review and Editing: PT, HS, AR, KK, SP-S. Figure Preparation: PT and SP-S. All authors contributed to the article and approved the submitted version.

## Funding

This work was funded by the Academy of Finland (SP-S, grant number 324238), Sigrid Jusélius Foundation (SP-S) and Päivikki and Sakari Sohlberg Foundation (PT).

## Conflict of Interest

The authors declare that the research was conducted in the absence of any commercial or financial relationships that could be construed as a potential conflict of interest.

## Publisher’s Note

All claims expressed in this article are solely those of the authors and do not necessarily represent those of their affiliated organizations, or those of the publisher, the editors and the reviewers. Any product that may be evaluated in this article, or claim that may be made by its manufacturer, is not guaranteed or endorsed by the publisher.
